# Risk of Leaching in Soils Amended by Compost and Digestate from Municipal Solid Waste

**DOI:** 10.1155/2014/565174

**Published:** 2014-06-03

**Authors:** Marta García-Albacete, Ana M. Tarquis, M. Carmen Cartagena

**Affiliations:** ^1^Escuela Técnica Superior de Ingenieros Agrónomos, Universidad Politécnica de Madrid, Ciudad Universitaria sn, 28040 Madrid, Spain; ^2^CEIGRAM, Campus Sur de Prácticas de la E.T.S. Ingenieros Agrónomos, Universidad Politécnica de Madrid, Ciudad Universitaria, 28040 Madrid, Spain

## Abstract

New European directives have proposed the direct application of compost and digestate produced from municipal solid wastes as organic matter sources in agricultural soils. Therefore information about phosphorus leaching from these residues when they are applied to the soil is increasingly important. Leaching experiments were conducted to determine the P mobility in compost and digestate mixtures, supplying equivalent amounts to 100 kg P ha^−1^ to three different types of soils. The tests were performed in accordance with CEN/TS 14405:2004 analyzing the maximum dissolved reactive P and the kinetic rate in the leachate. P biowaste fractionation indicated that digestate has a higher level of available P than compost has. In contrast, P losses in leaching experiments with soil-compost mixtures were higher than in soil-digestate mixtures. For both wastes, there was no correlation between dissolved reactive P lost and the water soluble P. The interaction between soil and biowaste, the long experimentation time, and the volume of leachate obtained caused the waste's wettability to become an influential parameter in P leaching behavior. The overall conclusion is that kinetic data analysis provides valuable information concerning the sorption mechanism that can be used for predicting the large-scale behavior of soil systems.

## 1. Introduction

In the EU, between 118 and 138 million tons of biowaste are produced each year, approximately 88 million tons of which are municipal waste. This latter value is projected to increase by 10% by 2020. European standards encourage the recovery of the organic fraction of municipal solid waste (MSW) by composting, anaerobic digestion, or incineration [1], reducing the amount of waste sent to landfills in accordance with the Landfill Waste Directive [2]. In 2006, the EU was host to 124 plants performing anaerobic digestion of biowaste, with an overall treatment capacity of 3.9 million tons y^−1^ and a total annual compost production of 4.8 million tons.

The use of compost and digestate as soil fertilizer provides agronomic advantages, such as the improvement of several soil properties: structure, water infiltration, water-holding capacity, microorganism content (both amount and diversity), and nutrient content [3]. In particular, better phosphorus recycling may reduce the need for mineral fertilizers.

The most recent communication from the Commission to the Council and the European Parliament on future biowaste management steps in the European Union [1] proposed the recovery of both the digestate and compost from MSW anaerobic digestion as sources of stable degraded organic matter (approximately 45% of EU soils are characterized by low levels of humus [4], especially in southern Europe).

This new communication [1] promotes the digestate and the compost from MSW to be directly applied to the soil as valuable sources of organic matter and nutrients. Therefore, it is necessary to study the behavior and interaction of these biowastes with soils to evaluate their optimal use and potential environmental problems.

In many catchments around the world, agriculture is now the major contributor of P to surface waters [5]. The transport of P from agricultural soils to surface waters has been linked to eutrophication in fresh water and estuaries [6–8].

According to a recent document from the EU, “Sustainable phosphorus use” [9], 90% of the total phosphorus entering the food system (mineral fertilizer and organic manure) is lost before reaching the product, mostly via dissipation into the water system. Global losses from the soil to fresh water are estimated at 18.7 to 31.4 million tons per year [10]; in the EU-27, losses to leaching and runoff are estimated at 0.16 million tons per year [11].

Reuse of organic waste has been based on crop nitrogen (N) requirements and usually supplies P in excess of crop needs [12, 13]. Long-term phosphorus application to soils as fertilizer or manure can increase the potential for P loss to ground and surface waters.

These “excessive” soil P concentrations can be measured by environmental soil P-tests, such as water-soluble P (WSP) and FeO-P, which have been linked to P loss from agricultural land, or by agronomic soil tests, such as Mehlich-1 and Mehlich-3, which estimate the P available for crop growth [14–16]. The P source WSP test is a reliable mean for predicting the dissolved reactive P (DRP) concentrations in runoff from surface-applied manures and biosolids [17]. This phosphorus fraction should consist largely of the inorganic orthophosphate (PO_4_^3−^). The concentration of this fraction constitutes an index of the amount of phosphorus immediately available for algal growth. The WSP/TP ratio (the fraction of the total P that is water soluble) allows a more direct comparison of the environmentally relevant P in biosolids and manures with differing chemical and physical properties [18]. For leaching experiments, Sharpley and Moyer [19] found that the amount of P leached from six livestock manures and manure composts was significantly correlated with the water-extractable P in the materials (in the absence of soil). Other authors have suggested that the WSP of the P source materials is a good preliminary predictor for approximating the P leaching loss [20]; however, this may not account for continuous P release from added organic materials during continuous water infiltration.

Leaching and surface runoff experiments allow to evaluate the potential losses of soil P produced by the application of P sources to soils. Although P loss in runoff is considered to be the major contamination route, leaching also causes significant losses. Some authors have suggested that P leaching to groundwater is unimportant because the leaching is negligible [21]; however other authors report that the downward movement of P from organic wastes is potentially significant in areas with shallow groundwater and coarse soil with low P-absorption capacity [22, 23].

Laboratory leaching tests are common tools for assessing the long-term impact of contaminated materials on the soil-groundwater pathway by determining the source term as an expression of the release potential of water-soluble contaminants during the use or disposal of waste materials. These tests provide a flow-through pattern similar to that found in field conditions and permit the basic characterization of waste materials [24]. The release of soluble components upon contact with water is regarded as a main mechanism of release, and this result in a potential risk to the environment during the reuse or disposal of such materials.

Although the soil characteristics are not generally considered to be important in surface runoff tests [25], soil type is relevant in leaching tests due to its effects on the behavior of P, especially in applications by incorporation. The soil adsorption index of P (PSI) was analyzed for its relationship to the P source losses.

The evaluation of the P speciation in biowaste is very important when determining the suitability of biowastes for land application or the optimum application rate.

The physical properties of biowastes are also important. The wettability of biowastes is an important property in the leaching process in soil. Several authors have observed the influence of soil wettability in aggregate stability and the decomposition of soil organic matter [26, 27]. Although strong water repellency has been shown to have negative effects on hydrological process (e.g., soil erosion), a slight increase in water repellency may reduce the breakdown of aggregates and consequently reduce surface sealing, overland flow, and erosion. Hydrophobicity, as a measure of water repellency, caused by organic substances, favors the formation and protection of stable aggregates [28] which, in turn, stabilize the encapsulated organic substances against microbial degradation and mineralization [29, 30].

Wettability may have an important effect on the stabilization of SOM due to a reduction of liquid adsorption rates, accessibility for microorganisms, and restricted accessibility of water and nutrients. Hydrophobic SOM is more stable against microbial decomposition.

In soluble species in the waste that could be lost by leaching, water must pass through the soil profile, wet the waste, and dissolve these species. Soil water repellency has been extensively studied and is mainly caused by organic compounds of various origins and structures [31]. The wettability of soil particles increases with the charge density and fraction of polar groups on the surface [32, 33]. Sorption of organic matter with nonpolar functional groups promotes nonwettable surfaces [34], and long-chain amphiphilic organic compounds produced by a range of biota can induce hydrophobicity in soil [35]. When wetted, these compounds are usually hydrophilic, but drying can cause bonding of hydrophilic (polar) ends of amphiphilic molecules to each other or to particle surfaces, resulting in the exposure of hydrophobic (nonpolar) functional groups to the pore space [36]. This effect can be observed on biowastes after the application of various treatments.

The goals of this work were (a) to quantify the phosphorus leaching losses from organic waste generated in MSW treatment plants and (b) to evaluate the differences in the behaviors of digestate and compost on the potential mobility and P availability in three soil types. To that end, a first-order kinetic model was used to estimate digestate and compost wettability and leaching behavior.

## 2. Materials and Methods

### 2.1. P Source Samples

The biowastes applied as P sources were a compost and a digestate from an anaerobic MSW digestion plant, located in southern Madrid. Ten samples (5 kg each) were collected for the residues on different days and combined to produce a single homogenous sample representing each residue.

The digestate was obtained after the anaerobic digestion (21 d, 39°C, and constant agitation) of the organic fraction of MSW followed by dehydration with the addition of flocculants and centrifugation.

Compost was obtained by the composting in tunnels (14 d, 55°C, periodic watering and forced aeration) of a mixture of digestate of MSW and defibered plant matter, with a subsequent static stabilization period and sieving.

The digestate had a pasty texture, forming large clumps, whereas the compost was a powder with a much smaller particle size (<5 mm).

### 2.2. Chemical Analyses

The study was conducted with the surface horizon (0–20 cm) of three soils, from Madrid (40° 32′ N, 3° 17′ W), Guadalajara (40° 28′ N, 4° 0′ W), and Ciudad Real (39° 0′ N, 3° 56′ W) (Spain). All soil samples were collected in plots from agricultural research stations. These locations were chosen because their histories of P application are well known. The soils selected had not suffered applications of phosphorus in the last five years.

Soil organic matter (SOM) was determined by dry combustion at 540°C for 4 h. Soil pH was determined in a 1 : 5 (v/v) water extract. Soil Olsen-P was extracted with 0.5 M NaHCO_3_, pH 8.5, for 30 min and analyzed using the Murphy and Riley [37] spectrophotometric method. Calcium (Ca) and iron (Fe) concentration in soil were determined by inductively coupled plasma atomic optical emission spectroscopy (ICP-OES) following USEPA Method 3050A [38] acid digestion (with additions of nitric acid and hydrogen peroxide). The adsorption index of P (PSI) of soils was determined using the method of Bache and Williams [39] as the amount of P adsorbed (*X* in mg kg^−1^) after a single addition of a KH_2_PO_4_ solution containing 75 mg P L^−1^ divided by the logarithm of the P concentration in the equilibrium solution (Ce, mg L^−1^). OM content, pH, Olsen-P, and calcium (Ca) and iron (Fe) concentrations in compost and digestate were determined with the method described for soils. Total P (TP) levels of compost and digestate were determined from the same extracts used for Ca and Fe sampling. The total solids (TS) contents of compost and digestate were determined by preweighing the subsamples and drying in an oven at 105°C for 24 h. Kjeldahl nitrogen of both materials was determined by acid digestion and distillation over a solution of sodium hydroxide, followed by a back titration. Electrical conductivity (EC) was determined in the same 1 : 5 (v/v) water extract used for pH determination. Soluble organic matter of compost and digestate was analyzed by permanganate oxidation and distillation.

The P chemistry of the materials was extensively characterized. The analysis included the inorganic (IP) and organic P (OP) [40] and the water-soluble P (WSP). IP was extracted with 1 mol L^−1^ HCl, shaken for 16 h, and centrifuged (2500 rpm). The supernatant was filtered (0.45 *μ*m filters), and the extract was collected for IP determination. The previous residue was reused to determine OP, after calcination (450°C, 3 h), extraction with 3.5 mol L^−1^ HCl, shaking for 16 h, and centrifugation. The supernatant was filtered (0.45 *μ*m filters), and the extract was collected for OP determination. WSP was extracted at a 1 : 250 solid-water ratio after 16 h of shaking (end-to-end shaking) followed by filtration (0.45 *μ*m filters).

A modification of the fractionation method of Hedley et al. [41] and Sui et al. [42] described by Huang et al. [43] was employed to extract empirically defined pools of P. The P fractions were designated as WSP, membrane-P, NaHCO_3_–P, NaOH–P, and HCl-P. Samples were sequentially extracted with deionized water (WSP), deionized water with an anion-exchangeable membrane (membrane-P), 0.5 mol L^−1^ NaHCO_3_ (pH 8.5) (NaHCO_3_-P), 1 mol L^−1^ NaOH (NaOH-P), and 1 mol L^−1^ HCl (HCl-P). Total P in filtrates of water, NaHCO_3_, and NaOH extracts were determined by digesting aliquots of filtrates in an autoclave at 103.5 kPa with acidified (NH_4_)_2_S_2_O_8_ [44]. P in all the extracts was analyzed by inductively coupled plasma atomic emission spectroscopy (ICP-OES) following USEPA Method 3050A digestion. Labile P includes the sum of IP and OP from water, resin, and NaHCO_3_ fractions, whereas refractory or unavailable P includes the remaining fractions. The procedure was performed in triplicate on each biowaste sample.

A Sigma force tensiometer and the Washburn technique for the wetting of porous solids were used to estimate the biowaste wettability, with the mass of adsorbed liquid (water, g) measured by weight difference every second for 20 minutes. Finally, biowaste particle size was determined by a granulometric analysis.

### 2.3. Column Leaching Experiment

The leaching tests were performed according to the European standard CEN 14405 : 2004 “Characterization of waste—Leaching behavior test—Up-flow percolation test” [45].

Thirty glass columns (6 cm in diameter × 21 cm in height) were prepared by adding a layer of 21 cm of each of the three types of topsoils (0–15 cm) from Spanish research centers: soil A (Calcic Haploxerept), soil B (Petrocalcic Palexeralf), and soil C (Typic Haploxeralf) (Soil Survey Staff, 2010). Columns were prepared in triplicate for each experiment. The soils were air-dried, crushed, sieved (<2 mm), and homogenized.

Each soil (600 g) was amended with different P sources at an application rate of 100 kg P ha^−1^ (25.5 g of compost and 11.6 g of digestate, resp.), which is considered too high for agricultural needs. Due to the widespread practice of fertilizer application based on N content, in general, these high rates of P are very common in the field. Appropriate controls of soils without waste application were included, and all treatments were performed in triplicate. The columns were carefully packed to avoid the formation of preferential water paths. Fiberglass was placed in the bottom and top of each column to prevent soil loss.

The columns were saturated with distilled water from the bottom upwards, and the saturation was balanced at room temperature for 72 h. The distilled water was then allowed to flow out of the bottoms of the columns. The leaching was transported continuously for 20 d with a constant flow of 22 mLh^−1^ induced by a peristaltic pump from the bottom of each column. The leachate was collected from the top of the columns. Sixteen stages of leachate per column were collected, with liquid-solid (L/S) ranges of 0.1 to 10 L kg^−1^.

Leaching fractions were analyzed for dissolved reactive phosphorus (DRP), determined from the filtered samples (0.45 *μ*m) without digestion. The P content was determined by ICP-OES.

### 2.4. Kinetic Model

A variety of kinetic equations including zero-, first-, and second-order, fractionation-power, and parabolic-diffusion and Elovich equations have commonly been employed over the years to describe the kinetics of soil chemical phenomena [46–48].

In the case of the phosphorus leaching experiment data, P leaching (P) was adjusted to a first-order kinetic model:

(1) $$\begin{array}{c} \text{P}={\text{P}}_{\max}\cdot \left(1-e^{\text{kl}t}\right). \end{array}$$

Here, P_max⁡_ (mgkg^−1^) is the maximum phosphorus leaching expected, kl (h^−1^) is the rate of phosphorus leaching, and *t* (h) is the time.

The behavior of biowaste wettability (W) as a function of time is expressed as follows:

(2) $$\begin{array}{c} \text{W}=\text{Mw}\cdot \left(1-e^{-\text{br}\cdot t}\right). \end{array}$$

Here, Mw (g) is the maximum wettability expected, br (s^−1^) is the adsorption rate, and *t* is the time (s).

### 2.5. Statistical Analysis

Analysis of variance (ANOVA) using the *F*-test at a significance level of 0.05 was performed to establish the possible significant differences between the mean values of leached phosphorus among the different treatments and soil types.

## 3. Results

Table 1 provides details of some characteristics of the soils used in this study. The main differences between the soils are the higher clay content of soil A (28%) relative to the other soils, which are sandy (*≈*70%). Soils A and B are slightly basic (pH 7.5–7.9), whereas soil C is acidic (pH 5.9). All three soils have low levels of organic matter. The Fe and Ca concentrations are higher for soil B (5.0% Ca, 1.4% Fe). Values of PSI indicate the retention capacity of P for each soil. Soil B has the highest P adsorption capacity (112 mg kg^−1^), and soils A and C have similar P adsorption capacities (30 and 27 mg kg^−1^, resp.).

A summary of the chemical properties of the digestate and compost used in the study is provided in Table 2.

Total solid content is higher in compost (81.9%) than in digestate (28.2%). The digestate has a lower OM content (45.3%) than the compost (62.0%). With respect to acidity, the compost is neutral (pH = 7.5), whereas the digestate is slightly basic (pH = 8.5). The TP concentration of the digestate (7.49 g kg^−1^) is about twice that of the compost (3.09 g kg^−1^). The TP concentration, obtained after the calcination and acid extraction of biowaste, is a useful overall indicator of pollution but provides no information about the solubility of P species, which depends on their chemical forms.

Table 2 shows that IP was higher than OP in both digestate (94%) and compost (91%). The Olsen-P and WSP are much higher for the digestate (55% and 49% of the TP) than for compost (20% and 22% of the TP). Significant differences were observed in the calcium content, which was 5 times lower for digestate than for compost (0.8% and 4.5% for digestate and compost, resp.). The hydrosoluble organic matter content was also lower for digestate than for compost. The analyzed biowastes exhibited low concentrations of heavy metals and trace elements (data not shown).

**Figure 1:**
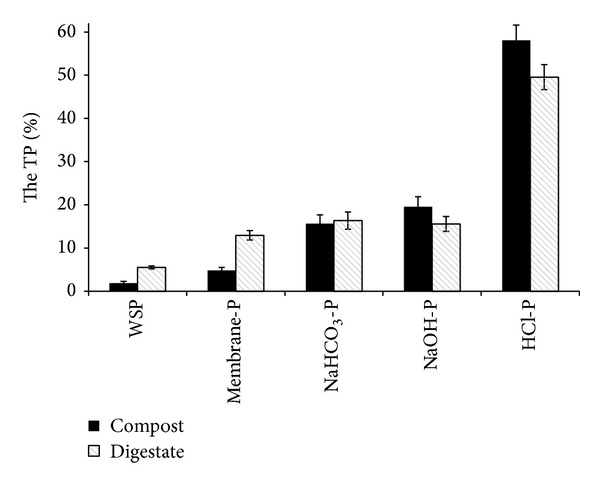
Fractionation of P in compost and digestate.

Percentages of WSP, membrane-P, NaHCO_3_-P, NaOH-P, and HCl-P with respect to total P for digestate and compost are given in Figure 1. The main P fraction for both biowastes was HCl-P, at 49.6% in digestate and 58.0% in compost, followed by NaHCO_3_-P (16.4%) in digestate and NaOH-P in compost (19.5%). WSP and membrane-P were the smallest fractions for both biowastes.

The sum of the percentages of TP composed of WSP and membrane-P, designated as “loosely bound-P”, is higher for digestate than for compost (18.5% and 6.7% of TP, resp.). The sum of the percentages of loosely bound-P and NaHCO_3_-P, designated as “labile P”, is also higher for digestate than for compost (34.8% and 22.4% of total P, resp.). The large amount of this fraction in digestate indicates high vulnerability for both P leaching and availability to plants.

Digestate has a much higher content of P that is easily lost. In contrast, the compost has high P content in the NaOH-P and HCl-P fractions, indicating that the extracted P is more recalcitrant and therefore more difficult to dispose of.

**Figure 2:**
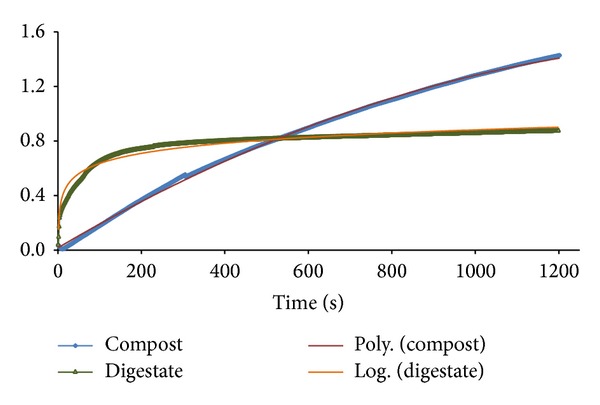
Time evolution of wettability of compost and digestate.

To characterize the wetting of each residue, Figure 2 shows the amount of water absorbed (g) versus time for the two biowastes studied, digestate and compost. The two residues exhibited totally different behaviors. Digestate initially absorbed water rapidly (up to 100 s); later, the amount of water absorbed was fairly constant, indicating a lower wettability. The compost absorbed a large amount of water relative to its weight, with water absorption increasing steadily throughout the trial (1200 s). Wettability results for digestate and compost were well described by a first-order kinetic model (Figure 2). The water adsorption rate (br) for digestate (17.9 · 10^−3^ s^−1^) was higher than for compost (1.02 10^−3^ s^−1^) but the maximum wettability (Mw) was much higher for compost (1.97 ± 0.09 g of adsorbed water per g of waste) than for digestate (0.82 ± 0.05 g of adsorbed water per g of waste).

**Figure 3:**
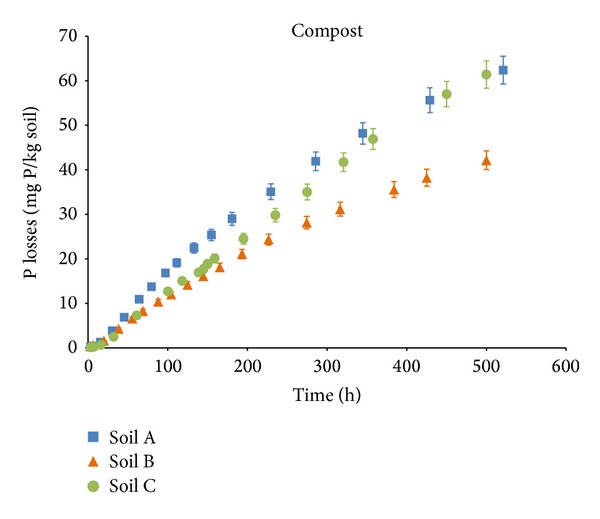
DRP losses by leaching in soil-compost mixtures. Error bars indicate the standard error of the mean (*n* = 3).

**Figure 4:**
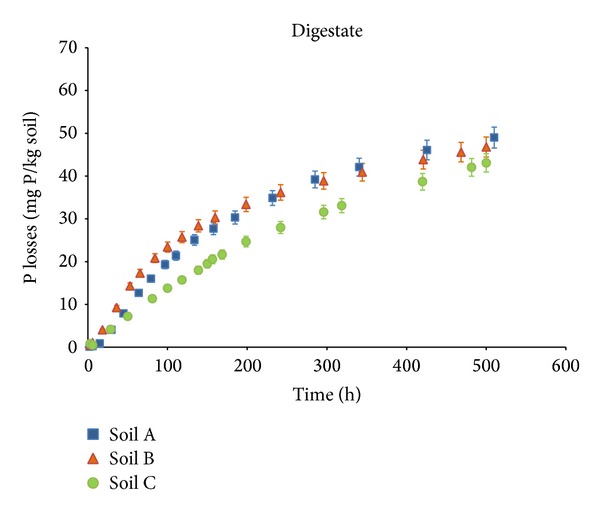
DRP losses by leaching in soil-digestate mixtures. Error bars indicate the standard error of the mean (*n* = 3).

Figures 3 and 4 represent the DRP (mg P kg^−1^ of soil) lost by leaching as a function of time (h) for digestate and compost in each soil. The total DRP concentration (mg P kg^−1^ of soil) accumulated in 10 L of leachate varies for each type of soil, from 43 ± 1.8 mg P kg^−1^ to 49 ± 1.5 mg P kg^−1^ for the mixtures of soil and digestate and from 42 ± 2.1 mg P kg^−1^ to 62 ± 2.3 mg P kg^−1^ for the mixtures of soil and compost. Losses of P vary from 33% to 37% and from 32% to 48% for digestate and compost, respectively.

Losses of DPR in columns amended with compost were more variable and lower for soil B than for soils A and C (Figure 3), which is consistent with the PSI values for each soil type. Soil B has the largest capacity to absorb P (112 mg kg^−1^) and thus a lower capacity for retention of P, while soils A and C have lower PSI (30 mg kg^−1^ and 27 mg kg^−1^, resp.). Results in columns amended with digestate were similar for the three soils (Figure 4). Columns of soil with compost, with a lower WSP content (22 % of the TP), lost large amounts of the P applied (from 32% to 47%). In contrast, the DRP losses from the columns treated with the digestate, which have a very high WSP content (49% of the TP), were lower than expected and varied less with the soil type (from 33% to 37%).

The dynamics of phosphorus leaching in the column experiments fitted a first-order kinetic model. Values of kl and P_max⁡_ are shown in Table 3. According to these results, leaching rates (kl) were higher for the soils with digestate (2.3 10^−3^ to 6.7 10^−3^ h^−1^) than for soils with compost (1.7 10^−3^ to 1.8 10^−3^ h^−1^).

In all cases, the calculated values for maximum phosphorus leached (P_max⁡_) were higher for soils with compost (72 to 108 mg kg^−1^). In soils amended with compost, P_max⁡_ values were significantly higher in soils A and C than in soil B (Table 3). This is consistent with the PSI values (Table 1) that showed lower absorption capacities in soils A and C.

Values of P_max⁡_ for digestate-amended soils ranged from 48 to 62 mg kg^−1^ and again were higher for soils A and C—those two having similar PSI—although the differences from soil B were not statistically significant (*α* = 0.05).

## 4. Discussion

Leaching of P from soils amended with MSW compost and digestate is a very complex process, involving many factors such as soil properties, waste characteristics, and water transport.

The application of these wastes produces interactions between P and soil components, depending on the physical and chemical properties of soils and wastes. Water-extractable P (WSP) and Olsen-P determinations are potentially useful to identify sources of P loss, but, in this experiment, none of these factors were correlated with P leaching from the digestate-soil and compost-soil mixtures.

The scientific community has agreed that characterization of P in biowastes is vital to finding indicators that provide significant information about the expected behavior of P when biowastes are applied to soils.

Our results indicate that considering only the biowaste WSP as an indicator of P leaching loss is not a good practice because there was no correlation between the WSP of each residue and the DRP content in the leachate (*r* ^2^ = 0.304). In almost all the experiments, the losses of P are higher in the compost-soil mixture than in the digestate-soil mixture, which is the opposite of the results expected from P fractionation analysis.

The behavior of the biowastes in wettability experiments can help to explain the results obtained in leaching columns. The digestate presents a lower wettability and consequently a lower interaction between the P in the digestate and the flow leachate. The compost, with a higher wettability, produces higher losses of P by leaching, although its WSP is lower. This demonstrates that the interaction between compost and water in the leaching columns is higher.

It is also important to note that compost has a larger overall surface area in contact with the water than the digestate, because the compost particle size is much smaller.

Kinetic data analysis aids our understanding of the sorption mechanism and prediction of the large-scale behavior of soil systems (Table 3). The rate of phosphorus leaching is similar for the three soils studied when compost is used as the amendment. In the case of digestate application, the constant rates are always higher than for compost, and they are more dependent on soil type. Lower losses were found for soil B (with the highest PSI) than for soil A and C (with similar, lower PSI).

## 5. Conclusions

As recent works have demonstrated, leaching losses of P by application of organic wastes cannot be neglected. Measuring biowaste for P indices (WPS, TP, or Olsen-P) that determine soluble or labile P are not useful for assessing P loss by leaching, though this is a practice currently applied in runoff experiments. Instead, it is necessary to evaluate other characteristics of the biowastes. In this experiment, waste wettability has been useful for explaining P losses.

The soil PSI is important for assessing potential P losses by leaching; however, the influence of soils is different for the two types of biowaste. The maximum phosphorus leached in soils amended with compost is significantly dependent on soils and consistent with the PSI values. In contrast, soils amended with digestate present lower total P losses, and the effect of soil is not significant. This phenomenon may be due to the lower soil-waste interaction as a consequence of digestate wettability.

Finally, our overall conclusion is that the P leaching rate depends mainly on the biowaste type and is less dependent on the soil type. However, the maximum amount of P leached depends on both the type of waste and the soil characteristics.

## Figures and Tables

**Table 1:** Chemical characteristics of the soils used in this study.

Parameters	Soil A	Soil B	Soil C
USDA classification	Calcic Haploxerepts	Petrocalcic Palexeralfs	Typic Haploxeralfs
Texture	Clay loam	Sandy clay loam	Sandy loam
Sand, %	55.0	70.4	71.0
Silt, %	17.0	8.0	11.0
Clay, %	28.0	21.6	18.0
pH, w extract 1 : 5	7.5	7.9	5.9
OM, %	1.41	2.22	1.03
Ca, %	0.7	5.0	0.06
Fe, %	1.1	1.4	0.6
Olsen-P, mg kg^−1^	18.8	17.9	10.1
PSI, mg kg^−1^	29.9	112.2	26.5

**Table 2:** Biowaste chemical characterization: compost and digestate.

Parameters*	Digestate	Compost
Particle size, mm	10–25	<5
Total solids, %	28.2	81.9
OM, %	45.3	62.0
Hydrosoluble OM, %	1.1	1.9
EC, dS m^−1^	6.8	4.7
PH	8.5	7.5
Kjeldahl-N, %	3.2	2.8
TP, g kg^−1^	7.49	3.09
WSP 1 : 250, g kg^−1^	3.64	0.69
IP, g kg^−1^	7.04	2.80
OP, g kg^−1^	0.39	0.25
Olsen-P, g kg^−1^	4.11	0.61
Ca, %	0.8	4.5
Fe, %	1.4	0.5

*Dry weight basis.

**Table 3:** First-order kinetic constant (kl) and P maximum leached estimated (P_max⁡_) for each biowaste and amended soil type.

BIOWASTE	Soil	kl 10^−3^ (h^−1^)	*P_max⁡_ (mg kg^−1^)
Compost	A	1.7	108.0^Aa^
	B	1.8	72.3^Ab^
	C	1.7	99.5^Aa^

Digestate	A	4.4	54.6^Ba^
	B	6.2	47.8^Ba^
	C	2.3	61.9^Ba^

*Same capital letters within a soil type indicate that there were no significant differences between the biowaste type at *α* = 0.05. Same lowercase letters within the same biowaste type indicate that there were no significant differences between soil types.

## Abbreviations

MSW: Municipal solid waste
WSP: Water-soluble phosphorus
DRP: Dissolved reactive phosphorus
TP: Total phosphorus
FeO-P: Phosphorus extracted by iron oxide
PSI: Soil adsorption index of phosphorus
TS: Total solid
OM: Organic matter
W: Wettability
Mw: Maximum wettability
Br: Biowaste rate
P_max⁡_: Maximum phosphorus leaching
kl: Rate of phosphorus leaching.

## References

[1] COM 235 final (2010). *European Commission*.

[3] Amlinger F, Nortcliff S, Weinfurtner K, Dreher P Applying compost—benefits and needs.

[4] Jones A, Panago P, Barcelo S

[5] OECD (2001). *Environmental Indicators For Agriculture*.

[6] Boesch DF, Brinsfield RB, Magnien RE (2001). Chesapeake bay eutrophication: scientific understanding, ecosystem restoration, and challenges for agriculture. *Journal of Environmental Quality*.

[7] Bush BJ, Austin NR (2001). Landscape and watershed processes: timing of phosphorus fertilizer application within an irrigation cycle for perennial pasture. *Journal of Environmental Quality*.

[8] Daniel TC, Sharpley AN, Lemunyon JL (1998). Agricultural phosphorus and eutrophication: a symposium overview. *Journal of Environmental Quality*.

[9] Science of Enviromental Policity (2013). *Sustainable Phosphorus Use*.

[10] Liu Y, Villalba G, Ayres RU, Schroder H (2008). Global phosphorus flows and environmental impacts from a consumption perspective. *Journal of Industrial Ecology*.

[11] Van Dijk KC, Lesschen JP, Ehlert PAI, Oenema O (2013). Present and future P use in the EU-27: food system scenario analyses. *Sustainable Phosphorus Use*.

[12] Sharpley AN, Herron S, Daniel T (2007). Overcoming the challenges of phosphorus-based management in poultry farming. *Journal of Soil and Water Conservation*.

[13] Huang X-L, Shenker M (2004). Water-soluble and solid-state speciation of phosphorus in stabilized sewage sludge. *Journal of Environmental Quality*.

[14] Sharpley AN, Robinson JS, Smith SJ (1995). Bioavailable phosphorus dynamics in agricultural soils and effects on water quality. *Geoderma*.

[15] Sims JT, Heckendorn SE (1991). *Methods of Analysis of the University of Delaware Soil Testing Laboratory*.

[16] Pote DH, Daniel TC, Sharpley AN, Moore PA, Edwards DR, Nichols DJ (1996). Relating extractable soil phosphorus to phosphorus losses in runoff. *Soil Science Society of America Journal*.

[17] Elliott HA, Brandt RC, O’Connor GA (2005). Runoff phosphorus losses from surface-applied biosolids. *Journal of Environmental Quality*.

[18] Brandt RC, Elliott HA, O’Connor GA (2004). Water-extractable phosphorus in biosolids: Implications for land-based recycling. *Water Environment Research*.

[19] Sharpley A, Moyer B (2000). Phosphorus forms in manure and compost and their release during simulated rainfall. *Journal of Environmental Quality*.

[20] Kang J, Amoozegar A, Hesterberg D, Osmond DL (2011). Phosphorus leaching in a sandy soil as affected by organic and inorganic fertilizer sources. *Geoderma*.

[21] Peterson AJ, Speth PE, Corey RB, Wright TW, Schlecht PL, Clapp CE (1994). Effect of twelve years of liquid digested sludge application on the soil phosphorus level. *Sewage Sludge: Land Utilization and the Environment*.

[22] Lu P, O’Connor GA (2001). Biosolids effects on phosphorus retention and release in some sandy Florida soils. *Journal of Environmental Quality*.

[23] Harris WG, Rhue RD, Kidder G, Brown RB, Littell R (1996). Phosphorus retention as related to morphology of sandy coastal plain soil materials. *Soil Science Society of America Journal*.

[24] Roussat N, Méhu J, Abdelghafour M, Brula P (2008). Leaching behaviour of hazardous demolition waste. *Waste Management*.

[25] Kleinman PJA, Sharpley AN (2003). Effect of broadcast manure on runoff phosphorus concentrations over successive rainfall events. *Journal of Environmental Quality*.

[26] Goebel M-O, Woche SK, Bachmann J (2012). Quantitative analysis of liquid penetration kinetics and slaking of aggregates as related to solid-liquid interfacial properties. *Journal of Hydrology*.

[27] Goebel M-O, Bachmann J, Woche SK, Fischer WR (2005). Soil wettability, aggregate stability, and the decomposition of soil organic matter. *Geoderma*.

[28] Sullivan LA (1990). Soil organic matter, air encapsulation and water-stable aggregation. *Journal of Soil Science*.

[29] Tisdall JM, Carter MR, Stewart BA (1996). Formation of soil aggregates and accumulation of soil organic matter. *Structure and Organic Matter Storage in Agriculture Soils*.

[30] Tisdall JM, Oades JM (1982). Organic matter and water-stable aggregates in soils. *Journal of Soil Science*.

[31] Doerr SH, Thomas AD (2000). The role of soil moisture in controlling water repellency: new evidence from forest soils in Portugal. *Journal of Hydrology*.

[32] Bachmann J, Goebel M-O, Woche SK (2013). Small-scale contact angle mapping on undisturbed soil surfaces. *Journal of Hydrology and Hydromechanics*.

[33] Bachmann J, Horton R, Ren T, Van Der Ploeg RR (2001). Comparison of the thermal properties of four wettable and four water-repellent soils. *Soil Science Society of America Journal*.

[34] Ellerbrock RH, Gerke HH, Bachmann J, Goebel M-O (2005). Composition of organic matter fractions for explaining wettability of three forest soils. *Soil Science Society of America Journal*.

[35] Capriel P, Beck T, Borchert H, Gronholz J, Zachmann G (1995). Hydrophobicity of the organic matter in arable soils. *Soil Biology and Biochemistry*.

[36] Hallett PD, Bachmann J, Czachor H, Urbanek E, Bin Zhang Z, Glinski J, Horabik J, Lipiec J (2010). Hydrophobicity of soil. *Encyclopedia of Agrophysics*.

[37] Murphy J, Riley JP (1962). A modified single solution method for the determination of phosphate in natural waters. *Analytica Chimica Acta*.

[38] USEPA (1995). *Process Design Manual: Land Application of Sewage Sludge and Domestic Septage*.

[39] Bache BW, Williams EG (1971). A phosphate sorption index for soils. *Journal of Soil Science*.

[40] Williams JDH, Mayer T, Nriagu JO (1980). Extractability of phosphorus from phosphate minerals common in soils and sediments. *Soil Science Society of America Journal*.

[41] Hedley MJ, Stewart JWB, Chauhan BS (1982). Changes in inorganic and organic soil phosphorus fractions induced by cultivation practices and by laboratory incubations. *Soil Science Society of America Journal*.

[42] Sui Y, Thompson ML, Shang C (1999). Fractionation of phosphorus in a Mollisol amended with biosolids. *Soil Science Society of America Journal*.

[43] Huang X-L, Chen Y, Shenker M (2008). Chemical fractionation of phosphorus in stabilized biosolids. *Journal of Environmental Quality*.

[44] Clesceri LS, Greenberg AE, Eaton AD (1998). *Standards Methods For the Examination of Water and wasteWater*.

[46] Ho YS, McKay G (1998). Sorption of dye from aqueous solution by peat. *Chemical Engineering Journal*.

[47] Raven KP, Hossner LR (1994). Soil phosphorus desorption kinetics and its relationship with plant growth. *Soil Science Society of America Journal*.

[48] Sparks DL (1989). *Kinetics of Soil Chemical Processes*.

